# Xenophobic Bullying and COVID-19: An Exploration Using Big Data and Qualitative Analysis

**DOI:** 10.3390/ijerph19084824

**Published:** 2022-04-15

**Authors:** Karla Dhungana Sainju, Huda Zaidi, Niti Mishra, Akosua Kuffour

**Affiliations:** 1 Faculty of Social Science and Humanities, Ontario Tech University, 2000 Simcoe St. N., Oshawa, ON L1G 0C5, Canada; huda.zaidi@ontariotechu.net (H.Z.); akosua.kuffour@ontariotechu.net (A.K.); 2 Rotman School of Management, University of Toronto, 105 St. George St., Toronto, ON M5S 3E, Canada; nitimkc@gmail.com

**Keywords:** xenophobic bullying, Twitter, COVID-19, social wellbeing, machine learning, qualitative analysis, misinformation

## Abstract

Extant literature suggests that xenophobic bullying is intensified by isolated national or global events; however, the analysis of such occurrences is methodologically limited to the use of self-reported data. Examining disclosures of racist bullying episodes enables us to contextualize various perspectives that are shared online and generate insights on how COVID-19 has exacerbated the issue. Moreover, understanding the rationale and characteristics present in xenophobic bullying may have important implications for our social wellbeing, mental health, and inclusiveness as a global community both in the short and long term. This study employs a mixed-method approach using Big Data techniques as well as qualitative analysis of xenophobic bullying disclosures on Twitter following the spread of COVID-19. The data suggests that about half of the sample represented xenophobic bullying. The qualitative analysis also found that 64% of xenophobic bullying-related tweets referred to occasions that perpetuated racist stereotypes. Relatedly, the rationale for almost 75% of xenophobic bullying incidents was due to being Chinese or Asian. The findings of this study, coupled with anti-hate reports from around the world, are used to suggest multipronged policy interventions and considerations of how social media sites such as Twitter can be used to curb the spread of misinformation and xenophobic bullying.

## 1. Introduction

The 2014 uniform definition for bullying identifies the behavior as an observed or perceived imbalance of power that is repeated or highly likely to be repeated. The definition also noted that bullying might inflict harm or distress on the target, including physical, psychological, social, or educational harm [1]. While most research on bullying addresses in-school or classroom behaviors [2,3], research on xenophobic bullying is limited but draws a wider focus by examining xenophobic bullying in broader contexts and circumstances [4]. The general parameters within which xenophobia is defined in the bullying literature consist of treatment resulting from the fear of a foreigner [5] or the ‘other’ [6]. The use of the word ‘fear’ is normalized for all segments of societal populations; therefore, research on xenophobic bullying stems beyond school-related bullying and addresses behavior in varying age groups [7]. In order to distinguish xenophobic bullying from other similar behaviors such as the prejudicial treatment of racialized people and/or discrimination, xenophobic bullying is largely classified as an emotional reaction aimed at specific individuals that are seen as a threat to the norms and values of the collective society [4,8].

The increase in xenophobic bullying is attributed to the occurrences of isolated or global events, which can foster an opinionated conversation online [9]. The worldwide outbreak of the COVID-19 virus is said to be among one of the most disruptive events to be recorded in modern history [10]. The first cases were identified in early January 2020 in Wuhan, China and the rapid spread of the virus worldwide led health officials to declare COVID-19 a pandemic on 11 March 2020. In addition to the obvious physical health concerns, the spread of COVID-19, much like previously recorded pandemics, exacerbated concerns of social and societal wellbeing [11]. The pandemic also ensued mass hysteria and panic with regard to its origin and spread. Having believed to have originated from China, most countries and online platforms saw a significant increase in xenophobic behavior, in particular, strong anti-Chinese and anti-Asian sentiment, which resulted in instances of anger, violence, and threats directed towards Asian communities and diaspora [12].

This study focuses on the first three months of the COVID-19 pandemic and, using Big Data, explores xenophobic bullying experiences shared on Twitter. The examination of the rhetoric within the first few months of the pandemic is particularly important and noteworthy since issues such as misinformation and fearmongering are most apparent during that time [13]. Other similar studies also opted to examine relevant data from the early months of the pandemic, noting that, as time progresses, negative connotations and xenophobic attitudes are likely to manifest in various ways, thereby reaffirming the importance of addressing such behaviors at the start of the pandemic [13,14]. Accordingly, using natural language processing, machine learning, and qualitative content analysis, the results from this study aim to generate insights into how the first weeks and months of the global COVID-19 pandemic fueled and exacerbated xenophobic bullying, particularly toward those of Asian descent or perceived to be of Asian descent. In contrast to the majority of studies that examined COVID-19-related xenophobic bullying, this study does not utilize solicited self-reported data; rather, by utilizing a methodological approach that addresses the limitations of self-reported data, it provides an interdisciplinary and novel approach that examines unprompted and unsolicited disclosures of COVID-19-related xenophobic bullying on Twitter.

Twitter is a microblogging and social networking site that allows users to communicate through 280 character posts known as “tweets.” Tweets can also include websites or hashtags, a metadata tag prefaced by the symbol #, which allows for easy indexing or cross-referencing. Twitter has approximately 396.5 million users worldwide, supports 33 languages, and over 70% of its user base is outside of the United States [15]. The worldwide scope of Twitter means that this study is also not limited to one geographic location and can tap into xenophobic bullying experiences shared from around the globe. Relatedly, according to Wachs et al. [16], online conversations with negative connotations towards a class of people also tend to deteriorate inclusivity while instigating negative social behaviors such as episodic racial bullying or alienating individuals or groups of people. This study utilizes Twitter to identify COVID-19-related “bullying traces”. Xu et al. [17] and Bellmore et al. [18] note that bullying participants utilize online platforms to share or respond to bullying experiences and these “bullying traces” allow us to gather various perspectives about both offline and online bullying episodes. The study allows us to expand our understanding of COVID-19-related xenophobic bullying traces and points to ways that methodologies such as natural language processing (NPL) and machine learning (ML) can be utilized to improve the online detection of abusive and xenophobic bullying content, especially as it relates to the pandemic. Moreover, understanding the rationale and characteristics present in xenophobic bullying from the beginning of the pandemic may have important implications for our social wellbeing, mental health, and inclusiveness as a global community both in the short and long term. As this pandemic continues to affect us worldwide, the findings from this study may help to address, prevent, or mitigate continuing xenophobic bullying as a result of COVID-19.

## 2. Literature Review

### 2.1. Xenophobia and Racist Bullying

The earliest forms of xenophobic behaviors can be observed in the interactions between those of various faiths before the start of the 19th century. More particularly, historical accounts of the time illustrate the fear and emotional reactions of Roman Catholics in their treatment of individuals of the Jewish faith, see [4]; however, followed by globalization and widespread migrations of tribes and people, xenophobic bullying and sentiment are not limited to religious differences [5,8]. Rather, according to more recent literature, xenophobia and xenophobic bullying, in particular, can be observed in many social settings such as schools and workplaces [19,20]. Moreover, with the progression of social media technologies, xenophobic bullying is now increasingly common and concerning for practitioners and policymakers [21]. Online xenophobic bullying mobilizes the victimization of individuals and groups beyond the confines of physical space and into the everyday lives of individuals on the world wide web [22]. Among some of the most common forms of xenophobic bullying is race-based bullying. In other words, victimizing someone based on their racial or ethnic identity. Like other forms of xenophobic bullying, the decision to bully a certain group or individual stems from a social identity perspective, according to which racially diverse individuals are seen as part of a salient ‘out-group’ [19].

While it is possible that not all individuals belonging to the perceived ‘out-group’ face stigmatization, the extant literature on xenophobic bullying in particular states that such individuals are far more likely to be bullied based on their belongingness to a certain racial or ethnic identity [22,23]. For example, a study on workplace bullying illustrated that employees that are foreign-born or immigrating from dissimilar countries are four times more likely to experience workplace bullying within the Swedish workforce [24]. Other studies with similar results indicated that racial or ethnic minorities in various workplace settings are seen as targets of workplace stress, thereby far more likely to be bullied in comparison to those belonging to or ascribing to the race of the majority [23,25]. Studies examining xenophobic bullying in schools also found similar results. Racial identities and gender identities continue to be a point of victimization for young adolescents, especially in middle school and secondary school [2]. Serious short-term and long-term consequences such as suicidal ideation and attempts, depression and other psychosocial health issues, and social isolation are commonly associated with xenophobic bullying in schools [3]. Furthermore, with the progression of social media technologies, practitioners and policymakers cited an increase in xenophobic bullying, often stemming from online platforms and resulting in further victimization of individuals in-person in schools [20].

### 2.2. Xenophobia and Racist Bullying Related to COVID-19

Global or newsworthy events accompanied by negative connotations towards a group or individuals can serve as a catalyst and exacerbate bullying both online and in person. The detrimental impacts of anti-Asian sentiment alongside the spread of the global COVID-19 pandemic presented individuals of Asian descent or those perceived to be of Asian descent with unique and unprecedented challenges. In addition to countless reports of physical assaults, anti-Asian sentiment also resulted in widespread xenophobic bullying, targeting not only individuals but also their culture, businesses, and overall livelihood [26]. Moreover, anti-Asian hate is not limited to one geographic location. The United States-based Stop AAPI Hate coalition received reports of 10,370 hate incidents against Asian American and Pacific Islanders (AAPI) between 19 March 2020 and 30 September 2021 [27]. Similarly, the United Kingdom-based organization End Violence and Racism against East and Southeast Asian Communities (EVR) reported that hate crimes against East and Southeast Asians in the United Kingdom rose by almost 50% in the last two years [28]. The Russian-based organization SOVA also reported an increase in attacks against those of Asian descent (SOVA).

Likewise, a study by Cheng [29] also illustrated how xenophobic perceptions held by those in the United States resulted in generational damages to the physical and mental health and wellbeing of Asian-Americans. The normalization of the use of terms such as ‘Wuhan flu’ or the ‘Chinese virus’, alongside other racial slurs by public figures such as heads of state and government officials, resulting from xenophobic bullying caused undeniable damage to the livelihood of Asian-Americans [13,30]. Individual and communal acceptance of and adherence to xenophobic bullying and propaganda resulted in the abandonment and boycott of Chinese businesses and communities, especially those located in areas commonly known as Asian-ethnic enclaves [29,31]. Other studies also cited significant increases in hate crimes directed towards Chinese communities, such as vandalism and the destruction of property see [32]. Cases of physical assault and/or bullying of Asian-Americans in commonly shared places such as public transport busses, grocery stores, and public sidewalks were also reported [33,34].

In addition to in-person instances of xenophobic bullying, there was and continues to be a significant increase in xenophobic bullying on online platforms [35]. Although the spread of hateful rhetoric and misinformation about the pandemic is not limited to online platforms, some studies suggested that the resultant online xenophobic bullying of individuals and communities has further jeopardized the health and wellbeing of affected individuals [36,37]. Additionally, online platforms were also pivotal in the spread of misinformation on the virus itself, which has led to an increase in anti-Asian rhetoric and xenophobic bullying [10]. For example, a study by Yang [38] examined the role of misinformation spread online. The author found that misinformation about Chinese culture, such as eating habits and cleanliness, began to surface as the spread of COVID-19 gained global momentum [38]. Such negative connotations wrongly associated with the Chinese culture on worldwide online platforms played an undeniable role in fueling anti-Asian rhetoric, which consequently led to widespread xenophobic bullying online [39].

### 2.3. The Current Study

The current study takes an interdisciplinary approach to examine a pressing and timely social issue; it examines bullying discourse on Twitter while emphasizing the rise of race-based bullying as a result of COVID-19. A few studies suggest that high-profile bullying incidents worldwide lead to an increase in bullying-related Twitter posts [17,40,41]. These posts come not just from those reporting the high-profile cases or defending the victims but also from current and former victims who tweeted to self-disclose their own online and offline bullying experiences. These disclosures are important since they indicate that while platforms such as Twitter may be conduits for bullying behavior, they also serve as therapeutic and cathartic means to interact, support, and share with others.

This study utilizes Twitter to identify COVID-19-related “bullying traces” [17,18]. The study focuses on the first three months of the COVID-19 outbreak, and we hypothesize that from the start of the pandemic, there was an immediate increase in xenophobic content, including hateful speech towards specific ethnic groups, especially Chinese people, since COVID-19 is believed to have originated from Wuhan, China. The study is a novel contribution to the literature as it utilizes a non-traditional source of data to examine COVID-19-related xenophobic bullying. A majority of previous studies and reports examining xenophobic bullying during COVID-19 rely on solicited and self-reported accounts such as surveys and interviews. While this has expanded our understanding of the prevalence and scope of pandemic-related xenophobic bullying, this approach is subject to the limitations present in all self-reported data, which may reduce the reliability and validity of the results. This can include response bias such as social desirability bias where participants provide a socially acceptable answer rather than the truth, limitations due to differing interpretations or comprehension of the questions, and concerns related to recall where participants are not able to accurately recall past behaviors [42,43]. Scholars contended that self-reported data related to bullying behaviors may be particularly susceptible to these limitations since not all bullies may admit to their negative behaviors [44], and there may be challenges with recall in retrospective studies that ask participants to think back to bullying events going back more than a year [45]. Additionally, there may also be issues due to sampling bias and variations in bullying definitions and participant understanding [46].

The current study addresses these limitations through the use of Big Data. The public nature of Twitter allows us to gather data on xenophobic bullying experiences that are unprompted and unsolicited. Moreover, the study allows us to tap into bullying experiences in real-time from individuals around the world using a random sampling strategy. The global nature of the COVID-19 pandemic would suggest that Twitter users from all around the world would utilize the platform to perpetuate, disclose, and share their personal or known xenophobic bullying episodes. Before the pandemic, 330 million monthly active users worldwide were using Twitter. Given that the pandemic shut the world down and forced countries around the world to employ social distancing and lockdown measures, one could argue that social media became an even more important source of information, communication, and socialization. Using human-coded tweets and machine learning algorithms, this study looks to examine the role of the tweet author, the form of bullying mentioned in the tweet, and why the Twitter user posted about the bullying behavior. Furthermore, the qualitative content analysis provides a more nuanced look at the type of xenophobic bullying behaviors being referenced, the characteristics of the bullies and the victims, and the rationale for the xenophobic bullying.

## 3. Methods

### 3.1. Data Collection

The data used in the study was gathered via Twitter’s streaming Application Program Interface (API), which represents a free data retrieval system where users can identify a list of keywords and tweets that match the keywords are retrieved. The data for the current study includes tweets from 3 January 2020, when China first officially notified the World Health Organization (WHO) of the COVID-19 outbreak, until 31 March 2020, two and half weeks after the WHO declared COVID-19 a pandemic and most countries around the world had entered into lockdowns and seen a significant rise in the number of cases and deaths. Tweets were collected with the primary keywords “*bullied, bully, bullying, cyberbullied, cyberbully, and cyberbullying*” for the study time period. These tweets were then additionally filtered with the following secondary keywords: “*corona, COVID, COVID-19, COVID19, coronavirus, sarscov2, SARS-CoV-2, covididiot, covidiot, virus*” to limit the tweets to COVID-19-related bullying incidents. Only tweets that matched both a primary and secondary keyword were retained. Then, re-tweets, tweets with more than six hashtags, non-English tweets, and tweets that only included a website were removed to clean the dataset and remove spam accounts. After the keyword filtering and data cleanup, 65,887 tweets were retained and served as the tweets utilized for the current study. Following this, tweets were tokenized, and each token was tagged using NLTK’s part-of-speech (POS) tagger to identify the lexical category of the tweet [47]. Then, hashtags were converted to a single token, URLs and user mentions were replaced with placeholders, and each token was subsequently lemmatized based on their POS tag through NLTK’s WordNet Lemmatizer [48]. Next, the tweets were transformed into a TF-IDF matrix [49] using unigrams (one token) and bigrams (two consecutive tokens), and the TF-IDF matrix was utilized for the supervised machine learning model discussed below.

### 3.2. Identifying Bullying Traces

Following this, the tweets were classified as a “bullying trace” if the tweet author participated in or mentioned a discreet bullying episode. Similar to previous studies [18,40,41], tweets were taken at face value rather than abiding by traditional definitions of bullying, which often include repetition, imbalance of power, and intent [50]. The focus was on the tweet author’s identification and interpretation of a COVID-19-related bullying episode. Thus, any reference to a COVID-19-related bullying episode that was personally experienced or being shared by the tweet author was considered a bullying trace. Tweets were not considered a bullying trace if it was a news headline that was simply copied and pasted without any additional original content, tweets that referred to a bullying episode that may happen in the future, tweets that shared an opinion about bullying instead of an actual discrete episode, and tweets that sounded like bullying but was not defined explicitly as bullying by the tweet author. Once the tweets from all days were concatenated together, 5000 tweets were randomly selected without replacement for annotation. Using this random sampling strategy, every tweet had an equal probability of being chosen, and any given tweet could not be chosen twice. Then, the 5000 randomly selected tweets were labeled independently by two coders. Following the criteria noted above, seven tweets were marked as “not applicable” as they did not have anything to do with COVID-19-related bullying. Another 3009 tweets, or 60.26% of the labeled tweets, were identified as non-bullying traces based on the exclusion criteria above. Based on 1000 of the 4993 tweets (this excludes the tweets marked as not applicable), an interrater agreement or Cohen’s kappa of κ = 0.74 was calculated, and of the 4993 labeled tweets, 1984 or 39.74% were classified as COVID-19-related bullying traces.

To classify the tweets, the study utilized validated and frequently utilized standard machine learning and natural language processing methods, including logistic regression and support vector machines (SVM) [17,51]. Eighty percent of the human coded tweets were used as the training dataset to fit the model, while 20% of the tweets were held back and used as the test dataset to validate and test the final model [52]. Each model was trained using stratified 12-fold cross-validation, and parameter tuning was conducted to find the best performing model. A combination of the average accuracy and average F-1 score on the test set was used to choose the final model for each classification task. The performance of the final models based on the test dataset is highlighted in Table 1.

### 3.3. Characteristics of COVID-19-Related Bullying Traces

Once the tweets were identified as COVID-related bullying and non-bullying traces, the tweets were further coded to examine three research questions: (1) Who was posting about the COVID-related bullying episode? (2) What form of bullying was being mentioned or used in the tweet? Additionally, (3) Why was the Twitter user posting about the COVID-related bullying episode on Twitter? The two independent coders labeled the 1984 tweets that were identified as COVID-19-related bullying traces to be used as part of the machine learning models to examine the three questions noted above.

The role of the tweet author was classified using bullying roles identified by Salmivalli [53], Xu et al. [17], and Bellmore et al. [18]. An author was identified as a ‘*victim*’ if they tweeted about an episode where they were being bullied or were bullied in the past. A ‘*defender*’ was someone who stood up against a bully in the tweet. A ‘*reporter*’ shared information about a COVID-19-related bullying episode but was not involved, including as a bystander. An ‘*accuser*’ accused someone of bullying; however, it was not clear if they were a victim, defender, or had another role. Three additional roles were also coded, an ‘*assistant*’ who did not initiate the bullying behavior but assisted the bully, a ‘*reinforcer*’ who encouraged the bullying behavior but was not directly involved, and a ‘*bystander*’ was someone who witnessed or was present but was not involved in the bullying behavior. The number of assistants, reinforcers, and bystanders for the current study was very small, so they were combined into one group referred to as the ‘*other’.*

Several forms of bullying were also classified. Similar to the bullying traces, this was also taken at face value and the explicit information provided by the Twitter user. Tweets where the user defined any physical actions used to bully or hurt someone were labeled as physical bullying. Verbal bullying was classified if the use of words such as insults, teasing, taunting, etc., was used in the bullying incident, and cyber bullying was instances where the bully used digital tools, technology, or online platforms to engage in bullying. Bullying traces that did not mention a specific form of bullying, or if a general reference to “bullying” was made, were labeled as general bullying. Finally, bullying behavior that was explicitly stated as being based on a fear of, hatred towards, or discrimination towards a group of people or individuals from specific geographic locations was labeled as xenophobic bullying.

The reasons for posting about the COVID-19-related bullying episode were based on a previous study by Bellmore et al. [18]. This included an accusation where the Twitter user was accusing someone of engaging in bullying. In a report, the tweet author described a bullying episode they knew about. A self-disclosure post was where the author revealed themselves as the bully, victim, defender, assistant, or another role. In a denial post, the author denied participating in a bullying episode and a cyberbullying post represented a direct attack from a bully on a victim.

### 3.4. Qualitative Content Analysis

To gain a deeper understanding of the characteristics of xenophobic bullying, a qualitative content analysis was conducted on bullying traces categorized as xenophobic bullying. Content analysis allows researchers to quantify and analyze the presence and meaning of certain words, themes, and concepts within qualitative data. To begin, the study authors used a directed content analysis approach and reviewed the tweets classified as xenophobic bullying to identify key categories and create an initial coding scheme. During this process, several key themes were identified, including the relationship of the tweet author to the victim and/or the categorization of the victim, the relationship of the tweet author to the bully and/or the categorization of the bully, the type of xenophobic behaviors being referenced, and the rationale for the xenophobic behavior(s).

Following this, using the initial coding scheme, two of the study authors independently coded 25 randomly selected tweets for each category, and the labeled tweets were compared to examine the level of agreement. The coding categories were discussed at length, and new codes were added to ensure that the codes reflected all the themes within the tweets. After this recontextualization phase, a codebook with all of the categories and codes was finalized for the categorization phase [54]. To increase the reliability, two additional rounds of coding were conducted with 25 new randomly selected tweets for each category and round until the two study authors reached a level of agreement of 80% or higher. See Table 2 for the final list of categories and codes that were derived during the categorization process. Then, using the final coding scheme, a qualitative content analysis was conducted on a set of new 300 randomly selected bullying traces categorized as xenophobic bullying using the qualitative software program Dedoose [55]. The 300 tweets represent approximately 7% of the xenophobic bullying tweets. Given the exploratory nature and the random sampling utilized in the qualitative portion of the study, we believe this to be a sufficient sample size. Moreover, prior studies that used similar methodologies analyzed samples that represent as little as 1% of the total sample [41,56]. After the content analysis, all the coded tweets were reviewed to ensure agreement with the coding. The 300 tweets served as the final content analysis sample, and the results are highlighted below.

## 4. Results

This study aimed to examine COVID-19-related xenophobic bullying Twitter posts to analyze “bullying traces” or online responses to bullying. We hypothesized that the COVID-19 pandemic would lead to an increase in xenophobic content, and this would be reflected in the bullying experiences shared by Twitter users online. Results of the machine learning models are discussed first, followed by the results of the qualitative content analysis.

### 4.1. Machine Learning Results

***Who was posting about the COVID-related racist bullying episodes?***

The tweets labeled by the human coders found that the most common tweet author was a reporter (38.03%), followed by an accuser (30.58%), defender (19.71%), victim (7.25%), and other (5.43%). The machine learning model achieved 56% accuracy. Compared to the accuracy of a naïve model that predicts the most frequent class, this represented an increase in skill. Similar to the human coded tweets, machine learning also found reporters to be the most common tweet author (61.92%), followed by accusers (28.32%), defenders (6.64%), victims (2.94%), and others (0.19%).

***What form of bullying was being mentioned or used in the tweet?***

The human coders found that general bullying (52.60%) was the most common form of bullying mentioned, which was followed by xenophobic bullying (39.68%). Cyberbullying was mentioned in 6.967% of tweets, and only a handful of tweets mentioned verbal bullying (0.61%) and physical bullying (0.15%). The machine learning model accuracy was 79% which represented a large increase in skill compared to the accuracy of the naïve model. The machine learning model predicted an almost equal distribution between xenophobic bullying (50.01%) and general bullying (49.47%) in the tweets. Cyberbullying represented less than one percent (0.52%), and there were no physical or verbal bullying tweets predicted. Both the human-coded tweets and the machine learning analysis supported our hypothesis as 40–50% of tweets mentioned xenophobic forms of bullying. Moreover, the most relevant keywords in identifying a COVID-19-related bullying trace in the machine learning model were the words “*Chinese*”, “*China*”, and “*Asian*”.

***Why was the Twitter user posting about the COVID-related bullying episode on Twitter?***

The human coded tweets found that accusations (44.47%) were the most common reason for posting, which was followed closely by reports (41.54%). A smaller number of tweets were posted to self-disclose (8.23%), cyberbully (3.79%), and deny involvement (1.97%). A 67% classifier accuracy was achieved by the machine learning model, which represented an improvement over the naïve model. The machine learning model predicted slightly more reports (53.46%) followed by accusations (42.74%). Similar to the human coded models, there were very few self-disclosures (3.54%), cyberbullying (0.17%), and denial tweets (0.09%). See Table 3 for a comparison of human coded versus machine learning predicted classifications.

### 4.2. Qualitative Content Analysis Results

***Who are the xenophobic bullying victims?***

**Figure 1:**
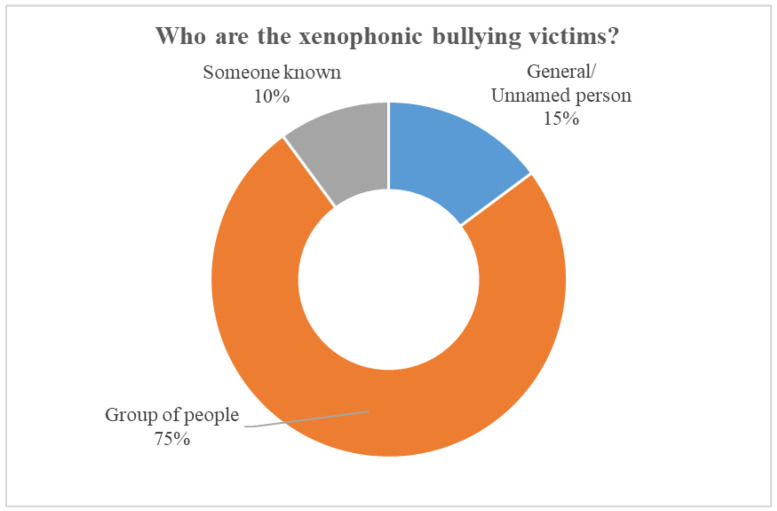
Xenophobic bullying victim characteristics.

The qualitative content analysis found that three-quarters (75%) of the victims referred to in the tweets represented a group of people. Corresponding to the most relevant keywords associated with identifying a COVID-19-related bullying trace in the machine learning analysis, the qualitative analysis also found that most often, the group of victims referred to people who were Chinese or Asian. For example, “*It is racist. Using such divisive language is unacceptable and dangerous. We already have stories of young Asian kids taunted and bullied in school, Asians assaulted and blamed for covid19. He is whistling to his base and it’s reckless*”. Another 15% were general, unnamed individuals, for instance, “*a kid is being bullied by his classmates here. kids telling him “go away corona virus, here comes corona virus*”! An additional 10% of tweets referred to a victim that was personally known to the tweet author or the tweet author themselves. For example, “*shut up you racist i literally get bullied in school and people say i have the c virus because i’m asian, so gross!!! god people like you literally makes me want me to die*”. See Figure 1 for more details on the xenophobic bullying victim characteristics.

***Who are the bullies engaging in xenophobic bullying or those perpetuating xenophobic bullying behaviors?***

**Figure 2:**
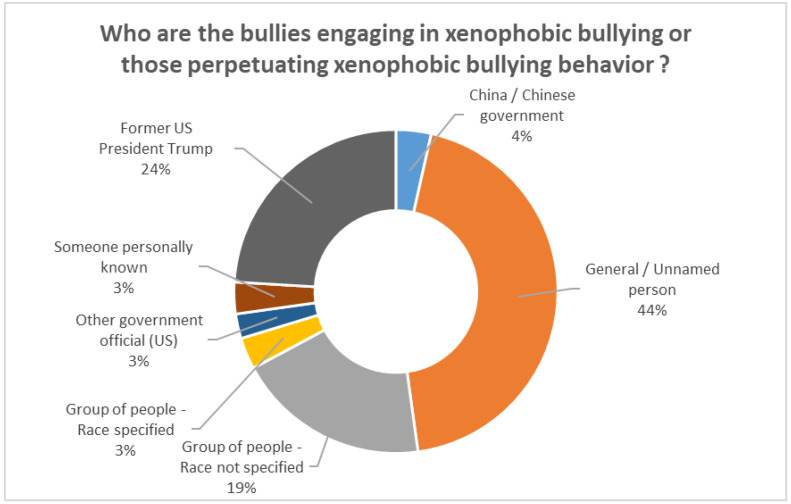
Xenophobic bully characteristics.

The tweets were examined to assess who was identified as the bully or who was being identified as perpetuating xenophobic bullying behaviors. A majority (44%) of the tweets did not specify a bully and were referring to a general, unnamed person. For example, “*my 6yo daughter got bullied at ymca daycare and we are not even chinese. i’ve never even been to china, not to mention her. They say “i’m not playing with you because you have coronavirus” and “i hate chinese people*”. Almost a quarter (24%) pointed to the rhetoric of former United States President Donald Trump and how it was perpetuating xenophobic bullying behaviors in tweets such as “*@realdonaldtrump stop, just stop!! your careless remarks are hurting the many children that are adopted from china. my granddaughter is being bullied just because she is Chinese*”. Within 19% of tweets, the bullies were identified as a group of people whose race was not specified, for example, “*asian kids are being bullied right now because their classmates think they have coronavirus, despite never going to china. think about what your words are saying and the impact it may have*”. A small percentage (4%) classified China or the Chinese government as the bully. Another 3% identified a group of people with their race specified as the bully, for example, “*i know i dont usually tweet personal stuff here but i just have to let this out. my cousin is literally getting bullied at school bc of this and white kids literally don’t want to sit with him and act like they’re gonna die when he coughed*”. Another 3% also identified the rhetoric of a US government official other than the former US President or an unnamed government official as perpetuating xenophobic bullying in tweets such as “*it would be nice if some US politicians would stop calling it Wuhan virus or China virus. Many Chinese overseas are being bullied for this*”. Finally, 3% of tweets referred to someone personally known as the xenophobic bullying perpetrator. See Figure 2 for the xenophobic bullying characteristics.

***What type of xenophobic behaviors were referenced in the tweets?***

**Figure 3:**
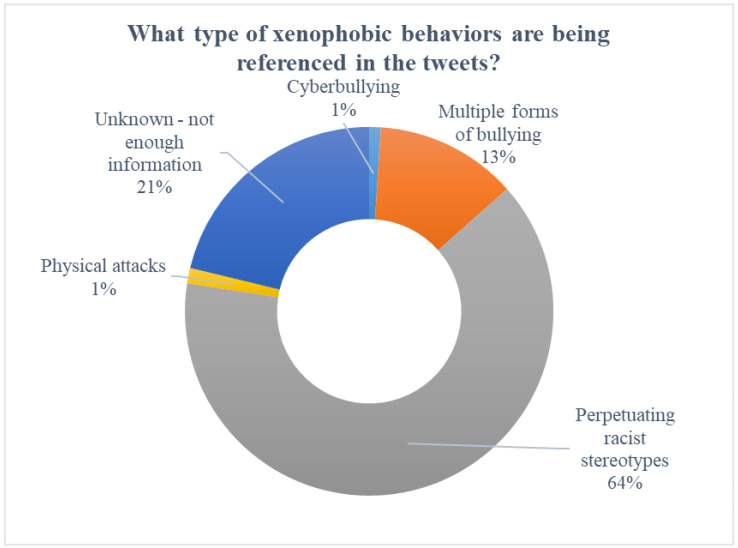
Type of xenophobic behaviors.

A majority of the tweets (64%) indicate that the bullies were perpetuating racist stereotypes. For example, “*i’ve already heard from three people who have been bullied because of their race in the last week. stand up to racism! it’s COVID or coronavoris, not chinese or wuhan virus. take care of people who are being attacked*”. In total, 21% of the tweets did not have enough information to ascertain the type of xenophobic behavior. A further 13% of tweets referred to multiple forms of bullying, for example “*there are more attacks against asians because of this! corona virus related crimes has become a regular term. elderly people are getting physically hurt by others and kids are being bullied at school worldwide*”! A small percentage of tweets referred to cyberbullying (1%) and physical bullying (1%). See Figure 3 for the types of xenophobic behaviors.

***What was the rationale for the xenophobic behavior?***

**Figure 4:**
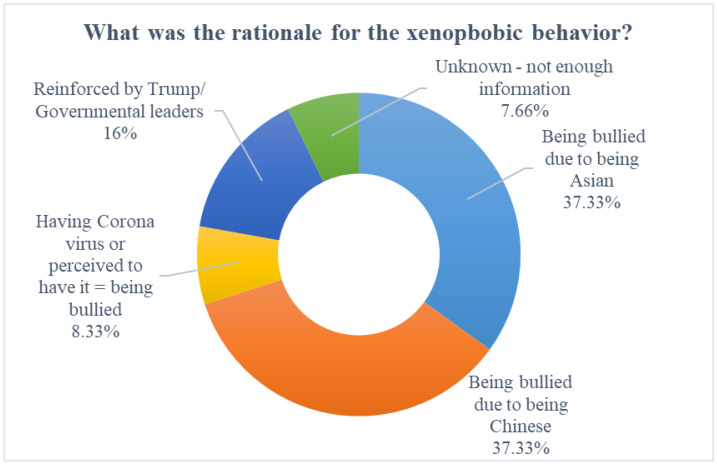
Rationale for xenophobic behavior.

Two main reasons for the xenophobic behaviors were identified from the tweets. The first was being bullied due to being Chinese (37.33%), as seen in tweets such as “*one of my daughter’s best friends, who is also chinese, has been bullied at her school about #coronavirus. for crying out loud, they’re only 10 years old!!! other kids must be getting these ideas from their parents. this disease is no excuse for #racism*”. The second was being bullied due to being Asian (37.33%), for example, “*I‘ve just heard from a friend her daughter got bullied in uk at school for being (half) asian, her classmates made a song about her and the corona. her daughter is devastated*”. Another 16% of tweets suggested that former U.S. President Trump or other government leaders were reinforcing xenophobic behaviors due to the use of xenophobic and stigmatizing terminology. In total, 8.33% of tweets were more general, suggesting that having the Coronavirus or being perceived to have it meant being bullied; for example, “*a school teacher witnessed a bunch of 7 year olds bullying and yelling “corona virus” at a Chinese boy for coughing. For this, I blame the parents. The worldwide sinophobia rn … not looking forward to the world my future kids will be born into*”. Lastly, 7.66% of tweets did not have enough information to indicate a rationale for xenophobic behaviors. See Figure 4 for the xenophobic behavior rationale.

## 5. Discussion

An examination of the first months of the COVID-19 pandemic sets the stage and helps us understand the roots of xenophobic bullying related to this worldwide event. It also allows us to consider the implications for our social wellbeing, mental health, and inclusiveness as a global community both in the short and long term. We hypothesized that the start of the pandemic would lead to an increase in xenophobic content, and the results support this hypothesis by illustrating how the first weeks and months of COVID-19 fueled and exacerbated xenophobic bullying, particularly towards those of Asian descent or perceived to be of Asian descent. The machine learning analysis found that a majority of tweet authors were reporters and accusers who were posting to accuse and report COVID-19-related bullying incidents. Although about half of the tweets did not directly mention xenophobic bullying and referred to bullying in more general terms, about 50% of tweets specifically referenced xenophobic bullying incidents. The qualitative analysis also found that 64% of xenophobic tweets referred to instances that perpetuated racist stereotypes, and the rationale for the behavior for almost 75% of xenophobic bullying incidents was due to the victim(s) being Chinese or Asian. Another reoccurring theme found in the qualitative analysis was the role that world leaders, in particular, former United States President Donald Trump, played in instigating and further exacerbating the spread of anti-Asian rhetoric. As seen in the example tweets, many tweet authors were pointing to the harm caused by the mainstream use of terms such as “China virus”, “Chinese virus”, “Kung flu”, or the “Wuhan virus”.

Our past can define our future and the start of the pandemic, and the results found in this study have implications for the broader Asian communities and diaspora worldwide. Similar to this study, Das et al.’s [14] analysis of Twitter data also found that during the first six months of the pandemic, there was an increase in cyberbullying. Likewise, another report found a 900% increase in hate speech on Twitter directed towards China and the Chinese and a 200% increase in traffic to hate sites and specific posts against Asians [57]. The same report also highlighted racist abuse against Asians and the blame placed on people of Asian origin for the spread of the COVID-19 virus. Unfortunately, despite knowing more about the COVID-19 virus, its’ timeline, and progression, the anger and blame directed towards Asians or those perceived to be Asians found in our results continues to this day. Statistics from the U.S. Federal Bureau of Investigations (FBI) reported that in 2020, the majority of hate crime victims were targeted due to their race, ethnicity, or ancestry, and there was a 73 percent increase in anti-Asian hate crimes [58]. Responding to the detrimental rise in anti-Asian racism and xenophobia, in May 2021, U.S. President Biden signed the COVID-19 Hate Crimes Act into law. The law emphasized the increase in violence against Asian Americans and gave state and local jurisdictions funds to conduct crime-reduction programs, allowed the Department of Justice to expedite the review of hate crimes related to COVID-19, and made reporting resources available online in multiple languages [59]. According to Vejmelka and Matkovic [60], the delay in censoring hateful content and xenophobic bullying resulted in irreparable damage to the overall solidarity of online communities. Moreover, the lack of protective measures to curb behaviors such as those highlighted in our findings from the early months of the pandemic has led to the ostracization of individuals and communities while furthering the victimization they may have already faced during in-person encounters [34].

On another note, research on traditional forms of bullying, such as school bullying during the COVID-19 pandemic, suggests that there may be a “silver lining”, with studies from the United States and Canada showing a reduction in bullying rates [61,62]. However, the rise in xenophobic bullying suggests that this is not the case for all youth. A survey conducted by Jueng et al. [63] found that 81.5% of Asian American youth reported being bullied or verbally harassed, and one in four faced shunning or social isolation due to the pandemic. Many of the tweets in the current study reflected this reality, noting the discrimination and harassment faced by Asian youth or those perceived to be Asian. Almost all schools moved to online learning at the start of the pandemic; however, once schools began to re-open, school districts across the United States began to notice a trend among Asian American youth. According to U.S federal data, one year after the pandemic in March 2021, more than 60% of Asian American youth opted for continued virtual learning, compared to only 19% of white youth [64]. The Washington Post also reported that many communities in places such as New York City, Chicago, and Fairfax County, Virginia, saw the lowest return rates of Asian American youth to in-person learning, with many families making this choice due to the fear that their child would experience race-based bullying at school [65]. Relatedly, even as the pandemic progressed, a report by the Rand Corporation found higher rates of hesitancy to send kids back to school for the 2021–2022 school year among families of color, including among Asian families [66]. While remote learning has its advantages, there could also be negative consequences and challenges for extended online learning. This is especially true for youth who are linguistically isolated if English is their second language and their parents or guardians do not speak sufficient English, they have limited access to a computer or internet, they have a learning disability, or if they are living in poverty and cannot access educational resources [67]. Alarmingly, a recent systematic review of studies from 11 countries worldwide also found that school closures and lockdowns related to COVID-19 were linked to adverse mental and physical health problems for children and adolescents [68]. As a society, considerations of how extended remote learning and how the increased rates of race-based bullying can impact Asian youth also need to be a serious priority.

The COVID-19 pandemic led to a dramatic rise in anti-Asian xenophobia and bias, and we must address the hate and harm that this has caused. As we wrap up year two of the COVID-19 pandemic and enter year three, people are experiencing pandemic rage, an emotional reaction to feelings of anger, frustration, and helplessness related to tensions caused by the pandemic [69]. Furthermore, anger was found to contribute to the spread of COVID-19 misinformation, with conservatives more likely to consider false claims to be “scientifically credible” [70]. Based on this study’s findings and the progression of anti-Asian hate throughout the pandemic, it is clear that multipronged targeted interventions are needed. Mainstream media and journalism outlets worldwide must consider the negative impact of misinformation and normalize stigmatizing terms such as “China virus” and “Wuhan flu,” especially when employed by world leaders and public figures. Relatedly, social media outlets such as Facebook, YouTube, and Twitter serve as regular news sources for many [71], thus also playing a key role in combating misinformation. Chou, Gaysnsky, and Vanderpool [72] recommend that these efforts need to go beyond just fact-checking and should include efforts to enhance the general public’s health and science literacy, highlight the tactics used by those who spread misinformation, verify the accounts of credible experts and organizations, and encourage cognitive reflection where the public improves their ability to discern the credibility of information that they read and/or share.

Additionally, as pointed out by Logie and Turan [73], to mitigate the stigma associated with COVID-19, we must address social inequities, including racism and xenophobia. These racial biases are often rooted in fear of the ‘other’ [6] or the ‘out-group’ [19] and were apparent in the tweets captured for this study, where users shared instances of individuals perpetuating racist stereotypes and using racial slurs against those who are Asians or those perceived to be Asian. Accordingly, cultural sensitivity and interventions to educate the general public are vital in breaking the racialization effect of COVID-19. This can include education on appropriate language. For example, in the United States, Congresswoman Judy Chu, who serves as the chair of the Congressional Asian Pacific Asian Caucus, released a toolkit to guide and encourage her fellow lawmakers to use culturally sensitive language when discussing China’s role in the pandemic [74]. Similar efforts could be enacted within other social institutions, including schools and workplaces, as those were identified in this study and previous studies as venues where xenophobic bullying takes place. Community resources to combat xenophobia and race-based bullying, both for the victims of race-based bullying and allies, are also needed. The American Psychological Association contends that it is imperative to provide mental health advocacy in the form of psychoeducational workshops, therapy, and support groups to Asian communities [75].

Furthermore, promoting equity and inclusiveness requires allyship, and this can be built through dialogue and advocacy. The current study highlights how platforms such as Twitter are being used to bring awareness to and confront anti-Asian hate and bullying by reporters and accusers. The use of trending hashtags (i.e., #StopAsianHate and #StopAAPIHate), re-tweets, and celebrity statements can all serve as a powerful tool to rally against xenophobia. For instance, in 2021, Korean pop group BTS released a statement on Twitter denouncing anti-Asian discrimination and hate crimes, and it was the most re-tweeted post on the platform in 2021 [76]. The global nature and real-time feature of Twitter allow for worldwide advocacy efforts to open up a dialogue between everyday users, celebrities, elected representatives, and organizations. In addition, studies point to digital platforms as being key tools to help promote, mobilize, and create a larger footprint for social movements [77,78]. The methodologies utilized in this study, including natural language processing (NPL) and machine learning (ML), suggest that researchers, policymakers, and practitioners can gain a broader understanding of xenophobic bullying worldwide by looking to sources beyond the traditional forms of self-reporting.

## 6. Limitations and Future Research

This study was able to provide insight into the first days and months of the pandemic and helped us understand the roots of COVID-19-related xenophobic bullying. However, the study also faced some limitations. The human coding for the machine learning process and the qualitative analysis all relied on interpreting the tweets at face value. The study analysis did not rely on traditional definitions of bullying behaviors, roles, and characteristics but rather assigned meaning based on the explicit content presented in the tweet. The study was also missing potentially relevant demographic information such as age, gender, racial background, and socio-economic status of the Twitter users. Without additional background context and tweet author information, the intent of the tweet and key bullying concepts may not have been fully captured. Relatedly, utilizing human coding runs the risk of potential bias based on the researcher’s own background and experiences; thus, we placed importance on obtaining a high level of interrater agreement for the machine learning coding, and all qualitatively coded tweets were reviewed for agreement. The captured tweets were also limited to the keywords identified by the study authors. While this captured a breadth of tweets related to COVID-19 xenophobic bullying, it did not include all disclosures or instances of xenophobic bullying. For instance, not all users will disclose and describe their xenophobic bullying experiences with the term “bullying” and instead could define it with terms such as “harassment,” “trolling,” “abuse”, or “mistreatment.” Given the scope of possible synonyms to describe similar experiences, future studies should explore ways to expand the keyword selection to capture a broader range of xenophobic bullying disclosures. This study was also limited to tweets written in English. Since Twitter support 33 languages, if country-specific or language-specific explorations of xenophobic bullying are of interest, researchers can also select keywords that reflect specific cultures or languages and utilize methodologies similar to this study.

From the machine learning modeling perspective, we also found that ground truth affects model performance. Big Data can provide larger sources of data compared to self-reported data, but it is also unstructured; therefore, having a clear definition of categories has an impact on what researchers aim to predict. Consequently, prior knowledge and data collected from self-reported data are crucial to informing Big Data analysis. Some of the machine learning models had a higher level of accuracy than others; thus, future studies should build improved classifiers by refining the categories analyzed in this study. Moreover, larger training size datasets can improve model performance. At the time of the data collection for this study, Twitter’s streaming API imposed a data limitation on how many tweets could be retrieved each day. In 2021, Twitter announced the launch of the Academic Research Product track, which will allow researchers free access to the full history of public conversation related to identified keywords [79]. This should allow future researchers to increase their sample size and explore more advanced ML models to capture the complexity of human language. In addition, word-embedding techniques based on deep neural networks can also extract semantic knowledge that the methods used in the current study cannot. Future researchers can also explore how additional features such as Twitter users’ account details could be explored to improve model performance. Lastly, the study relied solely on public tweets for the analysis. The inability to capture data from private accounts suggests that we may not have a full picture of the online disclosures regarding xenophobic bullying.

## 7. Conclusions

Over the years, an abundance of literature on bullying was amassed; however, a paucity of research remains on the topic of xenophobic bullying even though extant literature indicates a rise in such behaviors in physical and virtual social spaces, particularly in the presence of a noteworthy event. This study examined COVID-19-related tweets to analyze the disclosure of xenophobic bullying episodes. In contrast with the use of self-reported data, the use of multiple and interdisciplinary methods of analysis enabled us to examine unprompted and unsolicited disclosures of COVID-19-related xenophobic bullying on Twitter. The methodological design of this study allowed us to reconstruct and contextualize the experiences of a global community from various perspectives. The study points to ways that social scientists can use Big Data to complement the strengths and address the gaps in self-reported data. It also affords researchers three unique advantages: access to larger volumes with bigger sample sizes, data variety by being able to gather data from individuals worldwide, and data velocity, the speed at which data can be generated and gathered in real-time. The findings of this study, alongside the limited literature that has examined xenophobic behaviors related to COVID-19, can be advanced by researchers and policymakers to generate new insights and explore multipronged targeted interventions. Additionally, the results point to how social media sites such as Twitter can be used to address, prevent, or mitigate continuing xenophobic bullying as a result of COVID-19 as this pandemic continues to impact us worldwide.

## Figures and Tables

**Table 1:** Confusion matrix showing agreement and disagreement between human coding and machine learning models.

Human Coded	Machine Learning Predictions (* Using Test Dataset)					Accuracy Naïve Model	Accuracy Machine Learning Model	F1-Score Machine Learning Model
**Bullying Trace (*N* = 1498)**	**Yes**	**No**				60%	75%	74%
**Yes**	317	278						
**No**	101	802						
**Tweet Author (*N* = 395)**	**Accuser**	**Defender**	**Other**	**Reporter**	**Victim**	30%	56%	50%
Accuser	75	7	0	39	0			
Defender	37	0	0	30	0			
Other	3	0	0	18	0			
Reporter	27	0	0	123	0			
Victim	1	0	0	13	15			
**Form of Bullying (*N* = 397)**	**Cyber**	**General**	**Physical**	**Verbal**	**Xenophobia**	7%	79%	76%
Cyberbullying	0	27	0	0	1			
General	0	198	0	0	11			
Physical	0	1	0	0	0			
Verbal	0	1	0	0	1			
Xenophobia	0	41	0	0	116			
**Reason for Posting (*N* = 397)**	**Accusation**	**Cyberbullying**	**Denial**	**Report**	**Self-disclosure**	65%	67%	65%
Accusation	133	0	0	41	2			
Cyberbullying	10	0	0	4	1			
Denial	3	0	0	5	0			
Report	40	0	0	122	3			
Self-disclosure	2	0	0	19	12			

**Table 2:** Qualitative categories and codes.

Qualitative Category	Codes
Who are the xenophobic bullying victims?	A group of people Someone known or the tweet author themselves General/unnamed individual
Who are the bullies engaging in xenophobic bullying or those perpetuating xenophobic bullying behavior?	Someone personally known General/unnamed person Former United States President Donald Trump U.S. government official (non-Trump) China/Chinese government Group of people–race not specified Group of people–race specified
What type of xenophobic behaviors were referenced in the tweets?	Perpetuating racist stereotypes Physical attacks Cyberbullying Multiple forms of bullying Unknown–not enough information
What was the rationale for the xenophobic behavior?	Being bullied for being Chinese Being bullied for being Asian Former U.S. President Trump or other governmental leaders reinforcing xenophobic behaviors Having Coronavirus or perceived to have it = being bullied

**Table 3:** Comparison of human-coded and machine learning predicted COVID-19-related bullying tweets.

	Human Coded Tweets		Machine Learning Tweets	
	Count	Percentage	Count	Percentage
**Bullying Trace**				
Yes	1984	39.74%	6974	27.38%
No	3009	60.26%	18,493	72.62%
**Tweet Author**				
Accuser	603	30.58%	1975	28.32%
Defender	369	18.71%	463	6.64%
Other	107	5.43%	13	0.19%
Reporter	750	38.03%	4318	61.92%
Victim	143	7.25%	205	2.94%
**Form of Bullying**				
Cyberbullying	138	6.97%	36	0.52%
General	1042	52.60%	3450	49.47%
Physical	3	0.15%	0	0.00%
Verbal	12	0.61%	0	0.00%
Xenophobia	786	39.68%	3488	50.01%
**Reason for Posting**				
Accusation	881	44.47%	2981	42.74%
Cyberbullying	75	3.79%	12	0.17%
Denial	39	1.97%	6	0.09%
Report	823	41.54%	3728	53.46%
Self-disclosure	163	8.23%	247	3.54%
